# Transcriptional Regulation of Female and Male Flower Bud Initiation and Development in Pecan (*Carya illinoensis*)

**DOI:** 10.3390/plants12061378

**Published:** 2023-03-20

**Authors:** Yifei Xie, Zhiying Hou, Miao Shi, Qiaoyan Wang, Zhengfu Yang, Kean-Jin Lim, Zhengjia Wang

**Affiliations:** State Key Laboratory of Subtropical Silviculture, College of Forestry and Biotechnology, Zhejiang A&F University, Hangzhou 311300, Chinazafuyzf@163.com (Z.Y.)

**Keywords:** *Carya illinoensis*, flower bud transcriptome, flower bud differentiation, paraffin sectioning, pecan yield

## Abstract

Pecan (*Carya illinoensis*) nuts are delicious and rich in unsaturated fatty acids, which are beneficial for human health. Their yield is closely related to several factors, such as the ratio of female and male flowers. We sampled and paraffin-sectioned female and male flower buds for one year and determined the stages of initial flower bud differentiation, floral primordium formation, and pistil and stamen primordium formation. We then performed transcriptome sequencing on these stages. Our data analysis suggested that FLOWERING LOCUS T (FT) and SUPPRESSOR OF OVEREXPRESSION OF CONSTANS 1 play a role in flower bud differentiation. *J3* was highly expressed in the early stage of female flower buds and may play a role in regulating flower bud differentiation and flowering time. Genes such as *NF-YA1* and *STM* were expressed during male flower bud development. *NF-YA1* belongs to the NF-Y transcription factor family and may initiate downstream events leading to floral transformation. *STM* promoted the transformation of leaf buds to flower buds. *AP2* may have been involved in the establishment of floral meristem characteristics and the determination of floral organ characteristics. Our results lay a foundation for the control and subsequent regulation of female and male flower bud differentiation and yield improvement.

## 1. Introduction

Pecan (*Carya illinoensis*) is a deciduous tree belonging to the *Carya* genus that is tall and straight, and its wood can be used in the wood industry [1]. The most valuable parts of pecan are the large, flavorful nuts, which are rich in unsaturated fatty acids [2] such as oleic, linoleic, and linolenic acid, dietary fiber, protein, and minerals [3]. Studies have shown that the nut, as part of dietary intake, can reduce the incidence of tumor [4], hyperglycemia, and hyperlipidemia [5]. The content of unsaturated fatty acids increases as the pecan fruit progresses from slow growth to rapid expansion, hardening, grain growth, and ripening [6]. Fruit development begins with flower bud germination, and pecan fruit yield is closely related to the number of flowers and the flowering period of both female and male flowers [7].

Flower bud differentiation is an important stage in the growth and development of flowering plants; it is a sign of the transition from the physiological tissue state of leaf buds (vegetative growth) to the tissue state of flower buds (reproductive development) and then the formation of the embryonic morphology of the flower organ primordium [8]. It is a complex morphogenetic process, during which internal nutrients [9] and external environmental and genetic factors have certain influences. The process is a result of the expression of genetic material under specific environmental conditions and the combined action of adequate nutrients and regulatory substances [10]. In addition to genetic material, temperature, light, and water are important environmental factors that affect flower bud differentiation [11]. Under photoperiod induction, leaves can transmit light signals into a light-signaling response cascade, thereby promoting flowering [12]. Phytochromes (*PhyA*, *PhyB*, *PhyC*, *PhyD*, and *PhyE*) and cryptochromes (*CRY1* and *CRY2*) genes regulate photoperiods [13]. *PhyA* and *PhyB* have distinct functions in regulating flowering, with *PhyA* mutants flowering slightly later under long-day conditions, and *PhyB* inhibits flowering [14]. Overexpression of *CRY2* leads to early flowering, indicating that it can sense photoperiods [15].

Prior to differentiation of the floral primordium, plants must undergo vernalization (a period of low temperature) to form the floral primordium [16]. Vernalization regulatory genes, including *FLC* and *FLF*, have been cloned from Arabidopsis [17,18,19]. In vernalization-related mutants, the increased activity of the *FLF* gene led to delayed flowering. *FLF* gene transcription was downregulated during vernalization-dependent flowering, thereby promoting flowering. The *FLC* gene encoded a MADS domain protein that functions as a flowering repressor. Both late-flowering vernalization-responsive ecotypes and mutants had high and steady-state levels of *FLC* transcripts, and *FLC* transcript levels decreased during vernalization-promoted flowering, which promoted flowering. Thus, *FLF* and *FLC* are central to regulating the vernalization response. Two other genes related to vernalization, *VRN1* and *VRN2*, were found to maintain the repressive effect of *FLC* [18]. It was found that *SPL* transcription factor and the MADs-box family can regulate flowering time [20], and the *FLC* gene can control floral transformation [21].

Flower development mainly goes through the transformation from vegetative growth to reproductive growth, the formation of inflorescence meristem, and the decisive formation and maturation of floral organs [11]. Based on the morphological description, structural adaptability, and gene regulation of floral organs in angiosperms, scientists have proposed a variety of floral organ development models, namely the A, B, C, D, and E models [22,23]. The ABC model [24] was first proposed based on studies of floral organ mutants in plants such as Arabidopsis and snapdragon. The combination of genes A, B, and C determines four floral organs, namely tepals, petals, stamens, and carpels, in the concentric flower wheel. Studies have found that *FLORAL BINDING PROTEIN 7* (*FBP7*) and *FBP11* in petunia (*Petunia hybrida*) [25] and Arabidopsis *SEEDSTICK* (*STK*), *SHATTERPROOF1* (*SHP1*), and *SHP2* [26,27] genes determine the formation and development of ovules [27], and they were then listed as functional gene D. Another series of MADS genes [28], namely *SEPALATA* family genes (including *SEPALATA* 1, 2, and 3) [29], are also involved in determining the position of petals, stamens, and carpel in the Arabidopsis floral primordium [30] and are known as functional gene E. Studies have shown that a combination of different functional genes can transform floral organs [31]. Hence, the flower development model has been extended further to the ABCDE model. These five genes encode transcription factors that determine flower development, form different protein tetramers, and combine with DNA to affect the expression of various floral organ genes and determine the formation of floral organs [22]. Variation in any group of genes may lead to morphological abnormalities in flowers [31].

Pecans are monoecious, which pistillate and staminate flowers are on the same plant, cross-pollinated plants [32]. The flowering time starts from mid-late March to early May [33], and the flowering time of male and female flowers differs [7]. The male flowers are ternate catkin inflorescences, mostly growing in the middle and lower part of the short branch-like mixed naked bud. The female flowers are spikes, some of which are sessile, and grow directly on the inflorescence rachis. Massive male flower bud differentiation and excessive flowering consumption of tree nutrients affect female flower bud differentiation and fruit development [33,34]. Pecan yield is closely related to the number of female flowers, the ratio of female to male flowers, weather conditions, pollination [7], and other factors. In this study, we examine flower buds’ differentiation microscopically to distinguish the stages of bud differentiation and primordium, pistil, and stamen formation. By observing the internal anatomical structure of female and male flower bud differentiation, we can learn the developmental dynamics and grasp the differentiation period of female and male flower buds from the microscopic level. We go on to perform transcriptome sequencing and analysis of the relevant stages to explore the regulation of pecan’s flower bud differentiation, thereby further elucidating the mechanism of its flower formation. Our results provide a theoretical basis for flowering improvement and lay a foundation for manipulating the differentiation of male and female flower buds and increasing yield.

## 2. Results

### 2.1. The Differentiation of Female and Male Flower Buds

**Figure 1:**
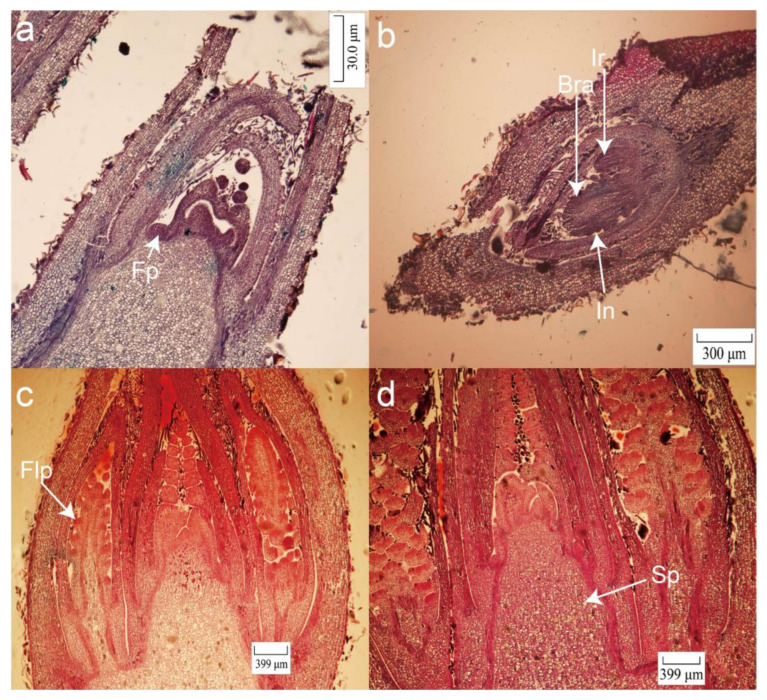
Morphological characteristics of pecan male flower bud differentiation. (**a**) The early stage of male flower bud differentiation (MD1) with flower primordia (Fp) can be observed in late April. (**b**) Male flower primordia with involucre (In), bract anlage (Bra), and inflorescence rachis (Ir) were observed from early until mid-May (MD2). (**c**) Floret primordia (Flp) also appeared in sequence with the elongation of the male inflorescence and increased the number of bracts in early July. (**d**) The stamen primordium (Sp) appeared in the middle of the male flower primordium depression after the calyx primordium differentiated in late July (MD3).

After the dormancy process in winter, male pecan flower and leaf buds began to germinate in mid- and late March. The new shoots began to elongate and grew in early April. In late April, the male flower buds were wrapped by 1–2 scales, and flower primordia could be seen. This stage was designated as the early stage of male flower buds (MD1, Figure 1a). We noticed that male flower buds differentiated into male flower primordia from early until mid-May. Involucre and rachis were also observed at this time (MD2, Figure 1b). We also observed bract primordia formed on the rachis with further development of the rachis. In early July, as time passed, the male inflorescence further differentiated, and there were floret primordium protrusions at the base of the scale-like bract tissues. The floret primordia appeared sequentially with the elongation of the male inflorescence and the increase in the number of bracts (Figure 1c). In mid-July, the male flower primordium flattened and began to be sunken, and the calyx primordium appeared at its edge. In late July, we noticed that stamen primordia appeared in the middle of the concavity of the male flower primordium (MD3, Figure 1d) about a week after the calyx primordia differentiated. After that, the stamen primordia were determined into anthers, and the columnar anthers were arranged side by side on the bracts. Bud development turned dormant in late autumn. In mid- and late March of the following year, the stamens began to expand and elongate, and anther sacs and pollen grains were formed.

**Figure 2:**
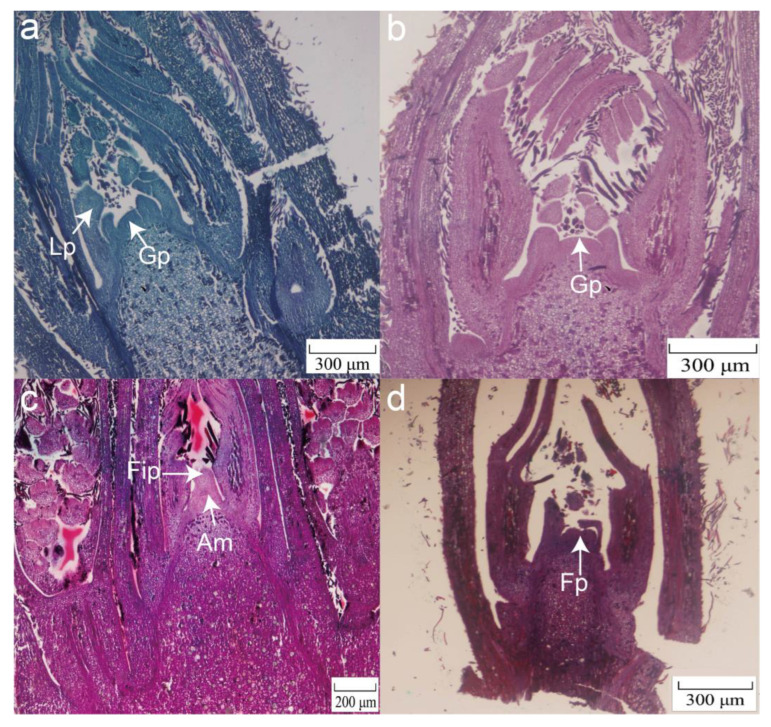
Morphological characteristics of pecan female flower bud differentiation. (**a**) The growing point (Gp) of the bud became smaller and pointed; the leaf primordium (Lp) began to differentiate in early May. (**b**) The flower buds gradually became flattened and sunken at the top growth point, and the flower buds entered the critical period of morphological differentiation, which lasted until the end of June (FD1). (**c**) From late June until mid-July, the growth point of the buds elongated upwards and gradually became rounded, forming a prominent spherical shape. The apical meristem (Am) and female inflorescence primordia (Fip) were observed during this period. (**d**) After the spherical protrusions were formed, the corresponding tips differentiated into the female flower primordia (Fp, FD2).

Female flower buds formed at the tip of the spring tip last spring and sprouted relatively later than the male flower buds. We noticed that in early May, the female flower buds’ growth point top became smaller and pointed and the leaf primordia began to differentiate (Figure 2a). As the number of nodes in the bud increased, that of the gradually differentiated leaf primordia increased. Thereafter, the apical growth point of the bud gradually became flat and concave. The flower bud then entered the morphological differentiation period, which would last until approximately the end of June (Figure 2b, FD1). After the critical period of morphological differentiation, the female flower bud’s growth point elongated upwards and gradually became rounded, forming a protruding spherical shape. The female flower inflorescence primordium could be seen during this period (Figure 2c). This stage was the female flower inflorescence formation period, which was roughly from late June to mid-July. After the spherical shape of the protrusion was formed, its lower part began to elongate and grow into the pedicel primordia. At the same time, the corresponding top was differentiated into the female flower primordium (Figure 2d, FD2). Female flower primordia and pedicel primordia appeared on different parts of the buds and lasted until between August and early September. Many pedicel primordia can form more than two female flower primordia simultaneously and develop into more than two female flowers in the future. After this, the bud differentiation entered a dormant state. The buds began to germinate from mid- to late March of the following year, and most female flower primordia differentiated into sepal primordia based on the upper end of perianth primordia, followed by petal and pistil primordia (FD3).

### 2.2. Transcriptome Data of Female and Male Flower Buds

Three differentiation stages of transcriptome data of female (FD1, FD2, and FD3) and male (MD1, MD2, and MD3) flower buds were obtained from Illumina paired-end sequencing. After quality filtering, we obtained a total of 106,7591,872 clean reads (Supplementary Table S1). Overall, we obtained an average of 44,482,995 clean reads per sample. Clean reads were mapped to the pecan genome with an average mapping rate of 90% (Supplementary Table S2). We validated the expression levels of several selected genes by quantitative real-time PCR (qPCR, Supplementary Table S3) of the female and male flower bud RNA samples that generated transcriptome data. These two datasets showed comparable results for the tested genes (Supplementary Figure S1).

### 2.3. Differential Expression Analysis of Female and Male Flower Buds

**Figure 3:**
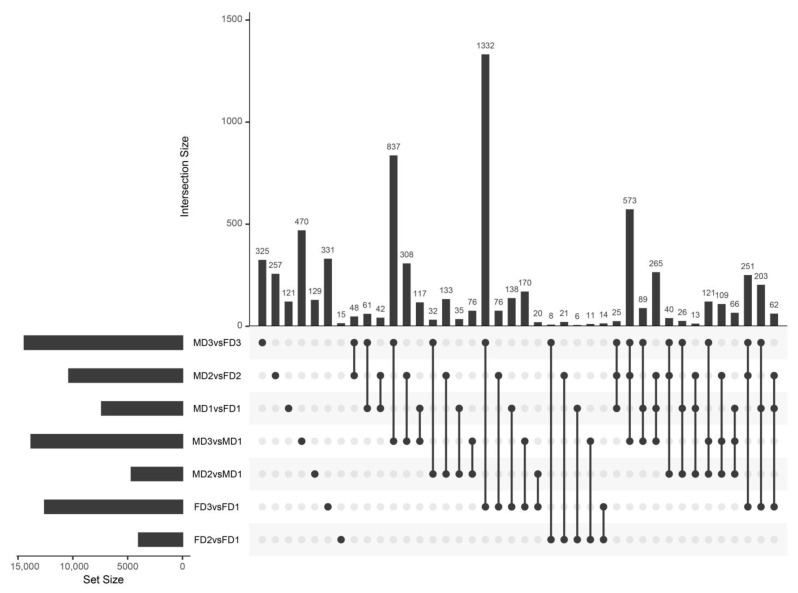
Overlap of differentially expressed genes between all comparisons. The horizontal and vertical bars of the UpSetR [35] plot indicate the number of differentially expressed genes in each comparison and the size of the gene set determined by only one comparison and intersection, respectively. Single nodes and corresponding horizontal bars represent the number of unique genes for this dataset and are not shared between other comparisons.

We performed pairwise differential expression analysis across all stages and between female and male flower buds to investigate transcriptome changes during female and male flower bud development (Supplementary Table S4). We noticed that the differential expression analysis of MD3 and FD3 returned the largest number of differentially expressed genes (DEGs), namely 14,418 DEGs (Supplementary Figure S2) and 325 unique DEGs (Figure 3), compared with pairwise comparisons of other male and female flower buds. The three comparisons of male and female flowers at the same stage had 25 common differential genes. In the female flower bud comparisons, FD2 versus FD1 had 15 specific differential genes, while in the male flower bud comparisons, MD2 versus MD1 had 129 specific differential genes (Figure 3). The UpSetR [35] analysis indicated that the number of shared differential genes in these comparison groups ranged from 6 to 1332, suggesting that some genes may have been expressed at specific or all stages during the development of the female and male flower buds (Figure 3).

### 2.4. Gene Set Analysis of Differentially Expressed Genes

**Figure 4:**
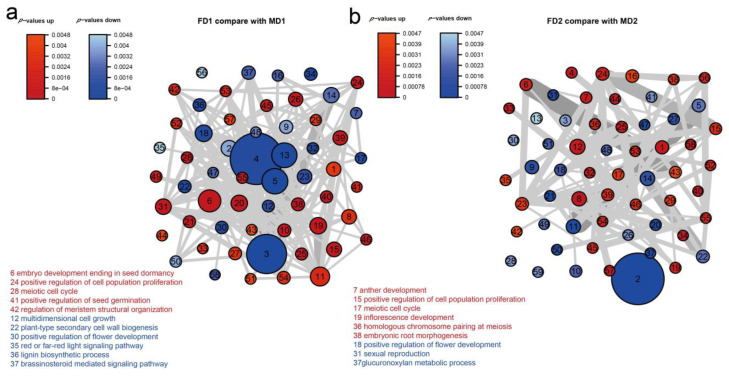
Gene set analysis of biological function changes during the development of female and male flower buds. (**a**) The initial differentiation stage of flower buds and (**b**) the flower primordial development formation stage. Biological function GO terms of differentially expressed genes of the early and second stages were subjected to analysis using R package PIANO [39]. The distinct directional network plot was drawn with a gene set significance threshold of *p* < 0.005. Each node’s size represents the node’s number of genes, and the width of the edges connecting nodes indicates the number of shared genes between nodes. The blue and red nodes represent the GO terms of differential genes of male and female flower buds.

We performed a gene set analysis (GSA) to learn transcriptome changes in biological functions during female and male flower bud development. The blue and red nodes represented the differential genes of male and female flower buds, as shown in Figure 4. A total of 7365 DEGs of female and male flower bud differentiation stages were analyzed (Figure 4a). In the initial stage of female flower bud development, the biological functions of “multidimensional cell growth”, “positive regulation of flower development”, “red or far-red light signaling pathway”, and “brassinosteroid mediated signaling pathway” and other biological functions were observed (Figure 4a, Supplementary Table S5). The results suggest that the photoperiodic flowering pathway was induced at this stage. These differential express genes regulate plant flowering, flowering time [36], and photoperiod-induced flowering [37]. During the early stage of male flower bud development, we noticed that the biological functions of “embryo development ending in seed dormancy”, “regulation of meristem structural organization”, and other biological functions were induced (Figure 4a, Supplementary Table S5). These functions involve the mitotic process or conversion of leaves into floral organs, and differentially expressed genes are associated with the floral transition [38].

During the female and male flower primordial formation stage, 10,361 DEGs were used for the analysis (Figure 4b, Supplementary Table S5). In the female flower bud development, biological functions such as “positive regulation of flower development”, “sexual reproduction”, and other biological functions were noticed. Genes related to the photoperiodic flowering pathway, or the sexual reproduction process were differentially expressed at this stage, indicating that the specific floral organs of female flowers were basically determined. In this stage of male flower bud development, biological functions such as “anther development”, “positive regulation of cell population proliferation”, “inflorescence development”, and other biological processes were observed (Figure 4b). We saw that genes associated with sex differentiation and anther and inflorescence development were differentially expressed in this stage. This suggests male flower-specific flower organs had been determined, with the same observation made in the second stage of female flower bud development (Supplementary Table S5).

### 2.5. Function Analysis of Differentially Expressed Genes

**Figure 5:**
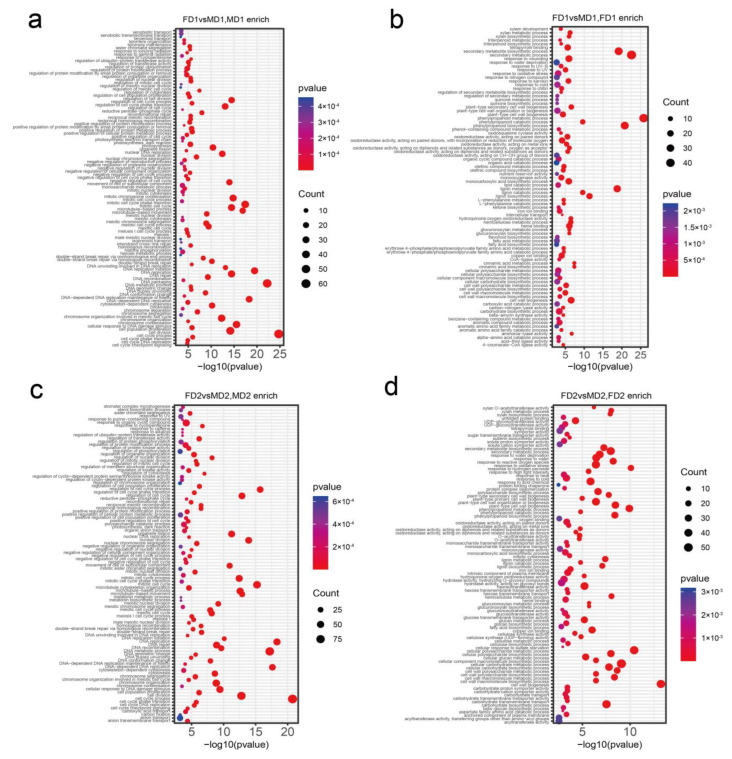
GO (biological process) enrichment analysis of initial differentiation of flower buds and the formation of flower primordial. The top 100 enriched GO terms were presented in each comparison. The enriched GO of the initial differentiation stage of (**a**) male (MD1) and (**b**) female (FD1) flower buds and the formation of the flower primordial stage of (**c**) male (MD2) and (**d**) female (FD2) flower buds. The vertical and horizontal axes represent the uniqueness of GO terms and enrichment factors, respectively. Scale bars are log_10_ *p*-values, and the size of circles represents the number of genes enriched in GO terms.

**Figure 6:**
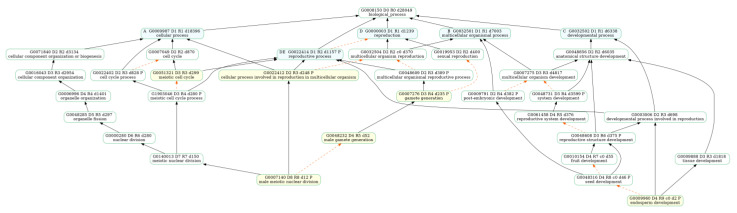
GOATOOLS [40] analysis of the hierarchical graphs of enriched GO biological processes in the early stage of male flower buds. Hierarchical relationships indicate that the resulting enriched GO terms (yellow) can be associated with reproductive processes (blue).

**Figure 7:**
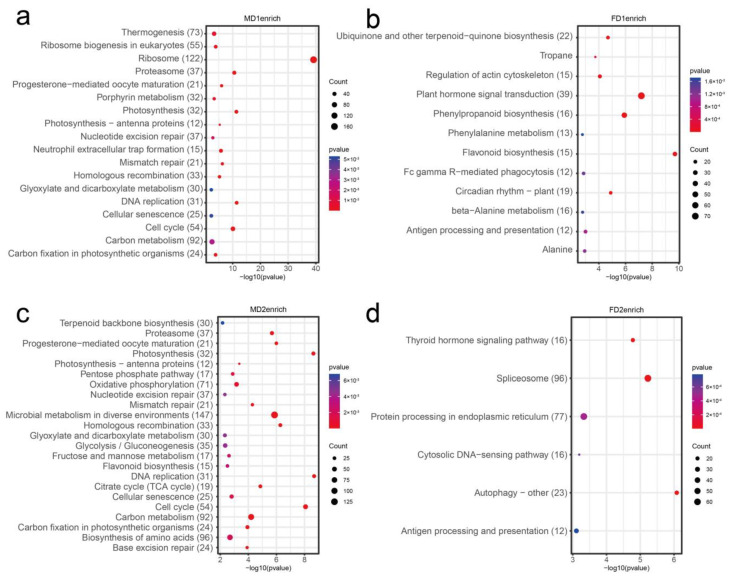
KEGG pathway analysis of flower bud initial differentiation and floral primordium formation. The enriched pathways of male (**a**,**c**) and female (**b**,**d**) flower buds in the initial differentiation stage (MD1, FD1) and formation of flower primordial stage (MD2, FD2), respectively. The vertical and horizontal axes indicate the enriched pathways and the enrichment factor uniqueness.

To understand the role of transcripts during the development of female and male flower buds, we performed gene ontology (GO) enrichment and Kyoto Encyclopedia of Genes and Genomes (KEGG) pathway analysis on DEGs at different stages (Supplementary Table S4). GO enrichment analysis of the early stages of male and female flower buds (FD1 vs. MD1) was compared, and we observed many terms related to male flower and reproductive development, such as “male meiotic nuclear fission”, “endosperm development”, “gametogenesis”, and “negative regulation of reproductive processes” were enriched on the male flower bud DGEs (Figure 5a; Supplementary Table S6). The hierarchical relational graphs (Supplementary Figure S3) show that these genes were indeed related to plant reproductive development (Figure 6). Genes related to cell growth and division, such as DNA replication origin binding, DNA repair complex, and microtubule-related genes (“microtubule-based movement” and “microtubule cytoskeleton organization”), were enriched at this stage. KEGG enrichment analysis revealed that the DEGs were mainly associated with “thermogenesis” and “DNA replication” (Figure 7a, Supplementary Table S6). Early-stage female flower bud DGEs were enriched with “cell wall biogenesis”, “plant type secondary cell wall biogenesis”, and “plant type cell wall biogenesis” which were related to metabolic processes (Figure 5b, Supplementary Table S7, Supplementary Figure S4). Moreover, at this stage, genes related to response to a stimulus, such as “response to UV-B”, “response to cold”, and “response to water deprivation” were also enriched. At the same time, KEGG analysis revealed that pathways associated with initial female differentiation, such as “flavonoid biosynthesis”, “plant hormone signal transduction”, and “beta-alanine metabolism” were enriched (Figure 7b, Supplementary Table S7).

During the formation of male flower bud primordia (Figure 5c), genes related to cell growth and division, such as “DNA replication origin binding”, “DNA repair complex”, and “DNA recombination” were enriched (Supplementary Table S8). They were associated with the developmental process (Supplementary Figure S5) and pathways of “flavonoid biosynthesis” and “cell cycle” (Figure 7c, Supplementary Table S8) from the KEGG analysis. On the other hand, in female flower bud primordia, genes related to cell wall synthesis, such as “cellular biosynthetic process” and “cell wall polysaccharide biosynthetic process” (Figure 5d, Supplementary Table S9), were enriched. They were related to establishing localization (Supplementary Figure S6) function. Pathways such as “spliceosome” and “protein processing in endoplasmic reticulum” were enriched (Figure 7d, Supplementary Table S9) at this stage.

**Figure 8:**
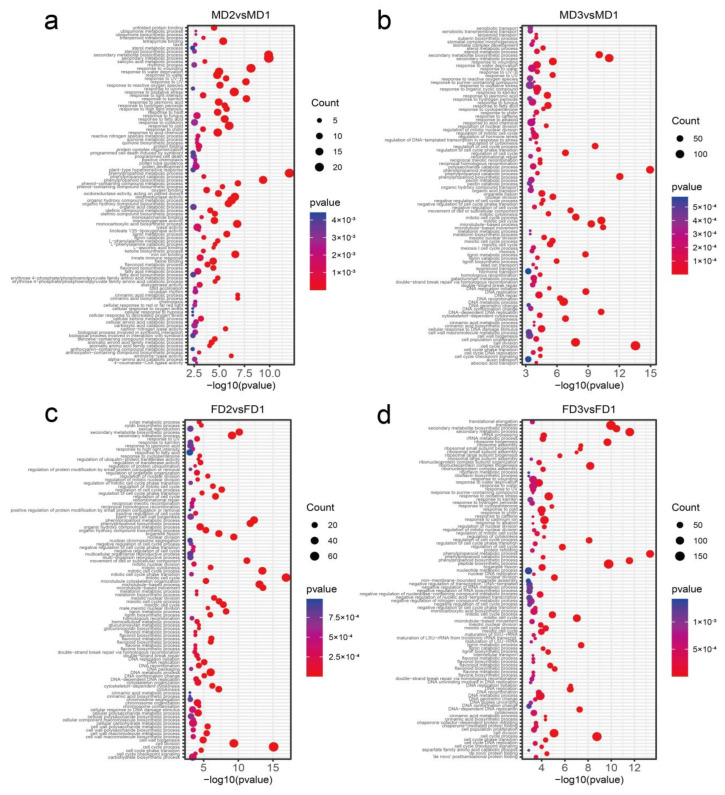
GO (biological process) enrichment analysis of differential genes in male and female flower buds development. The top row (**a**,**b**) shows GOs enriched in male flower buds at the formation of flower primordial (MD2) and stamen primordia (MD3) stages. Enriched GO for the formation of the flower primordial (FD2) and the pistil primordia (FD3) stages of female flower buds are shown in the bottom row (**c**,**d**). The top 100 enriched GO terms are presented in each comparison. The vertical and horizontal axes are enriched GO terms and the enrichment factor uniqueness, respectively. The unit of the scale bar is log_10_ *p*-value. The node size indicates the number of genes of the enriched GO terms.

**Figure 9:**
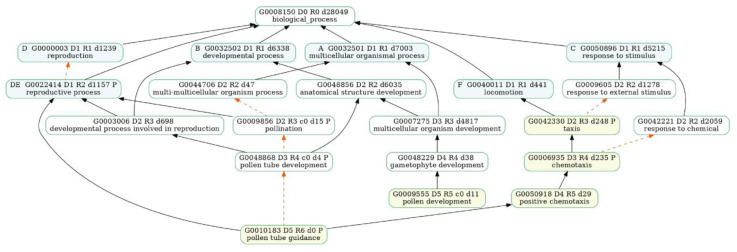
GOATOOLS [40] analysis of the association map of GO biological processes enriched in the second stage of male flower buds. Hierarchical relationships show that GO terms enriched in the results (yellow) can be associated with reproductive processes (blue).

**Figure 10:**
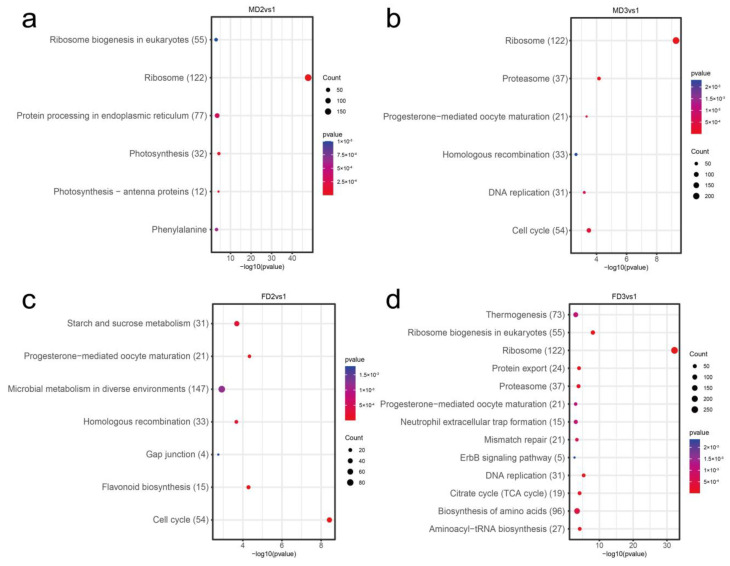
KEGG pathway analysis of male and female flower buds. The upper row shows enriched pathways for DGEs in male flower buds at the formation of the (**a**) flower primordial (MD2) and (**b**) stamen primordia (MD3) stages. The lower row indicates enriched pathways for female flower buds at the formation of the (**c**) flower primordial (FD2) and (**d**) pistil primordia (FD3). The enriched KEGG pathways and the uniqueness of enriched factors are shown on the vertical and horizontal axes.

We also looked at the ongoing biological processes during male flower bud development. We observed that the GO terms “pollen development” and “pollen tube guidance” and terms related to male flower organ development were enriched at the MD2 stage (Figure 8a, Supplementary Table S10), and they were associated with the development process, reproductive process, and locomotion (Figure 9, Supplementary Figure S7). KEGG analysis results were enriched in “ribosome” and “protein processing in endoplasmic reticulum” (Figure 10a, Supplementary Table S10), which related to development processes. At the MD3 stage (Figure 8b), in addition to genes enriched for reproductive development, genes responding to external environmental factors, such as “endosperm development”, “stomatal complex morphogenesis” and “stomatal complex development” were also enriched (Supplementary Table S11, Supplementary Figure S8). The pathways “cell cycle” and “DNA replication” were enriched, as we observed from the GO enrichment analysis (Figure 10b, Supplementary Table S11). 

**Figure 11:**
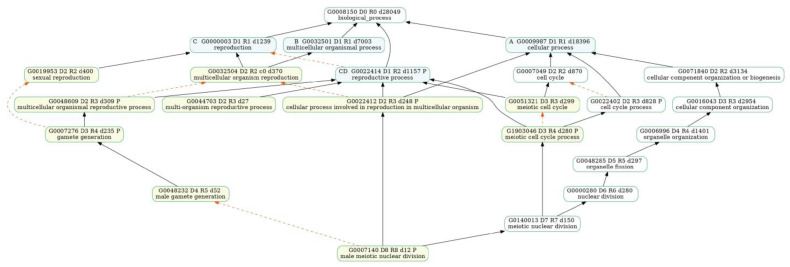
GOATOOLS [40] analysis of GO biological process association maps enriched in the second stage of female flower buds. The plot shows that GO terms enriched in the results (yellow) are associated with reproductive processes (blue).

Genes related to the reproductive process, such as “sexual reproduction”, “gamete generation” and “multi-organism reproductive process” were enriched at the FD2 stage (Figure 11, Supplementary Table S12). Moreover, cell wall-related terms such as “cell wall biogenesis” and “plant-type cell wall biogenesis” (Figure 8c, Supplementary Figure S9) were also significantly enriched. Some genes were also observed participating in the “progesterone-mediated oocyte maturation” pathway (Figure 10c, Supplementary Table S12), while at the FD3 stage, more GOs related to the cellular processes, such as “nuclear chromosome segregation” and “meiotic cell cycle” were enriched (Figure 8d, Supplementary Figure S10, Supplementary Table S13). The “thermogenesis” and “progesterone-mediated oocyte maturation” pathways were enriched at this stage (Figure 10d, Supplementary Table S13).

### 2.6. Gene Correlation and Expression Analysis

**Figure 12:**
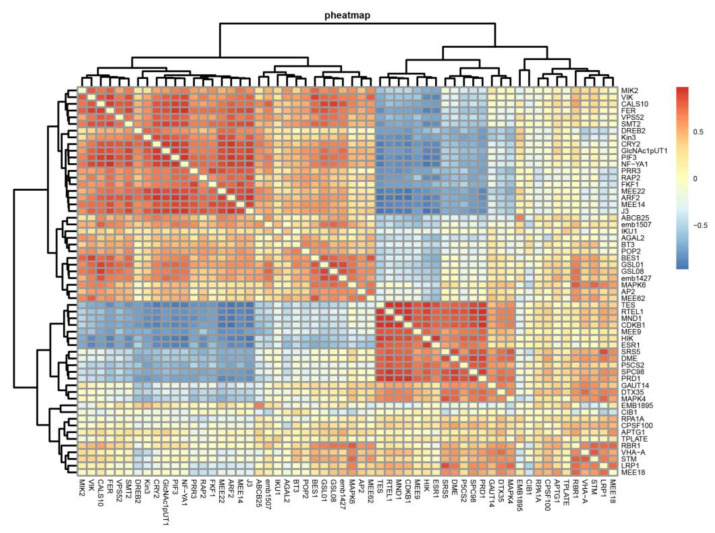
Correlation analysis of reproductive-related genes. The genes were divided into two clusters, and the genes in each group were positively correlated. Genes in one of the clusters were associated with photoperiodic flowering, brassinosteroid response pathway, and flowering time regulation. Another cluster was related to determining the transformation of leaf buds into flower buds in response to photoperiod and flower development. The correlation between genes was then calculated using the Pearson method of the *cor* function with log (TPM + 1) expression values. The scale bar unit was log TPM + 1.

We obtained 59 candidate genes related to the regulation of the reproductive development process (Supplementary Table S14) after examining genes related to flower bud differentiation and the sexual reproduction process through the analyses above. Correlation analysis divided these genes into two clusters (Figure 12). The groups of genes were positively correlated; however, the correlation between the groups was low. Genes *MIK2*, VH1-interacting kinase (VIK), *CALS10*, sterol methyltransferase 2 (SMT2), cryptochrome 2 (CRY2), nuclear factor Y-subunit A1(NF-YA1), auxin response factor 2 (ARF2), maternal effect embryo arrest 14 and 22 (MEE14, MEE22), DNAJ homologue 3 (J3), *GSL08*, and integrase-type DNA-binding superfamily protein (AP2), MAP kinase 6 (MAPK6) related to the photoperiodic flowering and brassinosteroid response pathways, and the regulation of flowering time [36] were grouped together and positively correlated. In contrast, genes *TES*, *MEE9*, *HIK*, Integrase-type DNA-binding superfamily protein (ESR1), *SRS5*, *DME*, *MAPK4*, cryptochrome-interacting basic-helix-loop-helix 1 (CIB1), *RTEL1*, Mnd1 family protein (MND1), *SPC98*, and KNOX/ELK homeobox transcription factor (STM) associated with determining the transformation of leaf buds into flower buds [38], responding to photoperiod [41] and flower development were positively correlated in another group.

**Figure 13:**
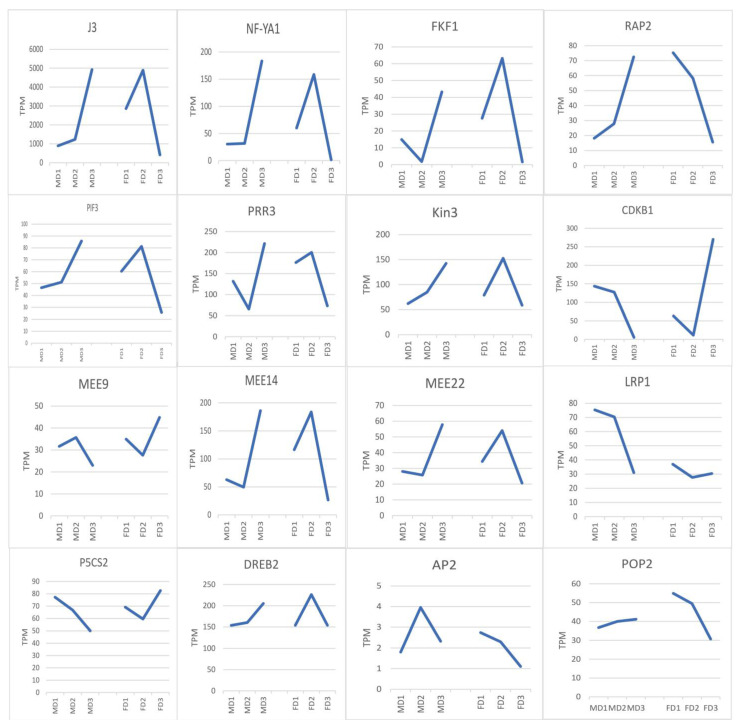
The expression trend of reproductive-related genes of male and female flower bud development in different stages. The expression trends of some genes were opposite during the development of male and female flower buds, suggesting that they may play different roles in the development of male and female flower buds. The vertical and horizontal axes are the average TPM values and male and female flower bud stages. TPM, transcripts per million; MD1, male flower buds’ initial differentiation stage; MD2, formation of male flower primordial stage; MD3, formation of the stamen primordia stage; FD1, female flower buds’ initial differentiation stage; FD2, formation of female flower primordial stage; FD3, formation of the pistil primordia stage.

We looked at the expression trends of these 59 genes (Supplementary Table S14) to shed light on their roles at different stages of female and male flower buds. Some genes showed opposite expression trends during female and male flower bud development, such as *P5CS2*, *DREB2*, *LRP1*, *MEE9*, *MEE14*, *MEE22*, *POP2*, *J3*, *RAP2*, *PRR3*, *PIF3*, *NF-YA1*, *Kin3*, *FKF1*, and *CDKB1* (Figure 13). These genes may play different roles in the development of female and male flower buds. On the other hand, some genes, such as *AP2*, *VIK*, and *GSL08*, shared similar expression trends, suggesting that they may have similar roles in female and male flower bud development.

## 3. Discussion

In this study, the developmental stages of flower buds were confirmed by sampling and sectioning female and male flower buds over one year. We then performed transcriptome sequencing on the initial differentiation of flower buds, the formation of floral primordia, and the formation of pistil and stamen primordia.

The male flower buds already had floral primordia at the early stage of differentiation (Figure 1a), but the female ones only had growth points (Figure 2a). We observed involucre and rachis flower organs emerging in the male flower buds during flower primordia formation (Figure 2b), which may be related to the difference in flowering time between female and male flowers [42]. Following analysis of the differential genes by using GSA, GO, and KEGG, the gene sets related to flower bud differentiation and flower organ development were observed. For example, “positive regulation of flower development” was enriched at the early stage of female flower differentiation and genes related to “inflorescence development” were enriched during the formation of male flower primordia.

Differential gene correlation analysis clustered the genes into two categories. One, mainly consisting of *STM2*, *CRY2*, *NF-YA1*, *ARF2*, *J3*, *AP2,* and *MAPK6*, was related to the photoperiodic flowering pathway [43], regulation of flowering time [36], and brassinosteroid response pathway [44]. Photoperiod-dependent flowering is one of several mechanisms by which plants initiate the transition from vegetative to reproductive growth [37]. CRY2 is a ubiquitous blue light receptor that establishes photomorphology and regulates flowering time [43]. AP2 consists of identifying floral organs and meristems, suppressing the uncertainty of floral meristems, and developing ovules and seed coats [45]. It was expressed at similar levels in female and male flower buds, and we hypothesize that it may play the same role in female and male flower bud development. 

The other cluster, consisting of *ESR1*, *CIB1*, and *STM*, was related to determining leaf buds into flower bud transformation [38] and responding to photoperiods [41] and flower development. *CIB1*, upregulated in female and male flower buds, interacts with *CRY2* to form a signal transduction pathway to activate floral initiation [41]. *STM* is a shoot apical meristem maintenance regulator that plays a role in transforming leaves into flower organs [38]. It was expressed in the early stage of the differentiation of pecan female and male flower buds and subsequently showed a downward trend. We speculate that it may determine the transition from leaf buds to flower buds in the early stage of flower bud differentiation.

Analysis of the expression of these genes in male and female flower buds at different periods showed that the high expression level of *J3* in the early stage of female flower bud differentiation played a role in regulating flowering time. *J3* mediates transcriptional regulation of floral pathway integrators, FLOWERING LOCUS T(FT) and SUPPRESSOR OF OVEREXPRESSION OF CONSTANS 1(SOC1), by interacting with the flowering repressor SHORT vegephase [36]. *NF-YA1* was significantly expressed in the early stage of male flower bud development. *NF-YA* is a part of the NUCLEAR FACTOR Y transcription factor, which can directly bind to the distal CCAAT box in the FT promoter, initiating a photoperiod-dependent flowering pathway [37]. At the shoot apex, the FT promoter can also activate its downstream targets, including Aptala 1 (AP1) and *SOC1*, which are required to establish and maintain floral meristem characteristics [46]. Phytochrome interacting factor 3 (PIF3) was up-regulated during male flower bud development, with the highest expression during stamen primordium formation. *PIF3* is a central regulator of plant growth and development and is involved in the regulation of pollen mitosis [47].

Some gene expression trends are opposite in the differentiation of female and male flower buds, such as that of detoxifying efflux carrier 35(DTX35). Deletion of its transcript affects the flavonoid level in plants, which will affect root growth, seed development, and germination, and pollen development, release, and survival [48]. Galacturonosyltransferase 14 (GAUT14) plays a role in pollen tube growth, possibly by participating in the biosynthesis of pectin in the pollen tube wall [49]. Some genes are maternal effect genes that function during the development of female flower buds, such as *MEE14* and *MEE9*, which are involved in the development and function of the female gametophyte [50] in flowering plants. MAPK6 encodes a MAP kinase induced by pathogenic bacteria, ethylene biosynthesis, oxidative stress, and osmotic stress and is also involved in ovule development [44]. 

We also observed that genes related to environmental factors such as response to water deprivation, cold, and UV-B were enriched in the female and male flower primordia. Temperature, light, and moisture [11] are important environmental factors affecting flower bud differentiation. Under the photoperiod induction, leaves can transmit signals by converting them into biological information and use regulatory means to promote flowering genes and repress the expression of the flowering factor [15,51]. Genes related to flavonoid synthesis, such as the flavone metabolism process, flavonoid biosynthesis process, and flavonol metabolism process, which help plants respond to abiotic stimuli and stress, were also significantly enriched [52].

## 4. Materials and Methods

### 4.1. Sample Collection

Male and female flower buds were collected from four pecan trees (cultivar Mahan) located in the orchard of Zhejiang A&F University (lat. 30°15′ N, long. 119°43′ E) in Zhejiang Province, China. The sample collection lasted for one year, from the beginning of April 2020 to the beginning of April 2021. The collected samples were used for microscopic observation and transcriptome sequencing.

Four female flower buds at the top of the fruiting branches were collected every three days from early April to early June 2020. The same number of female buds were collected at five-day intervals from mid-June to early August. The subsequent sampling was carried out with one monthly interval from early August to early March 2021, and after that, at three-day intervals from early March to early April 2021. The male flower buds were collected from the middle of fruiting branches. The sampling periods were the same as those described in female flower buds. 

The collected samples were immediately frozen with liquid nitrogen and stored in a −80 °C freezer for RNA isolation.

### 4.2. Microscopic Observation on the Morphology of Flower Bud Differentiation

Female and male flower bud samples were fixed and preserved with FAA solution (5% formalin, 5% glacial acetic acid, 50% or 70% ethanol, 50% ethanol for young buds, and 70% ethanol for hard buds). Fixed samples were then dehydrated by replacing water with a range of concentrations of ethanol. A range of concentrations of xylene was then used to replace the ethanol in the samples. The xylene in the samples was gradually replaced by paraffin wax until the samples was embedded in the wax. Hardened embedded samples were trimmed before sectioning. The trimmed samples were mounted on a microtome and cut into thin slices, each with a thickness of 8 µm. Thin sections were deparaffinized in xylene, ethanol, and water. This was followed by staining with 1% safranin solution and 0.5% fast green solution. After staining, the sectioned samples were dehydrated and placed in xylene then mounted on microscope slides using Canada balsam. The dried slides were observed under a BX60 OLYMPUS fluorescence microscope to determine the differentiation stages and photomicrographs of male and female flower buds. The paraffin methods had to be optimized due to the nature of the flower buds in this study. The optimization protocols are described in Supplementary Figure S11.

### 4.3. Extraction and Quality Control of RNA for Transcriptome Sequencing

Based on the observation of the paraffin sections, there were three differentiation stages of the female (F) and male (M) flower bud samples; the initial differentiation of the flower buds (FD1 and MD1), the formation of the flower primordial (FD2 and MD2), and the formation of the pistil (FD3) and stamen (MD3) primordia were used for transcriptome sequencing. Each stage contained four biological replicates.

The samples were ground using an RNAase-free mortar and pestle in liquid nitrogen. Total RNA was extracted from female and male flower bud samples using an RNA extraction kit (Tiangen, Beijing, China) according to the manufacturer’s protocol. The RNA purity and concentration were determined using a nanophotometer spectrophotometer (Thermo Fisher, Waltham, MA, USA), and the RNA integrity was determined with a Bioanalyzer 2100 (Agilent, Santa Clara, CA, USA). The qualified total RNAs were used for RNA sequencing library construction using the Illumina NEBNext^®^ UltraTM RNA Library Prep Kit according to the manufacturer’s protocols. The eligible RNA libraries were then subjected to 150 bp paired-end transcriptome sequencing (RNA-seq) using the Illumina NovaSeq platform.

### 4.4. Quality Inspection, Alignment, and Gene Expression Level Calculation

The low-quality and adapter contamination sequences were removed from the raw sequencing read using Trimmomatic [53] version 0.39 with the parameters of SLIDINGWINDOW:4:15 and ILLUMINACLIP:adaptor.fa:2:30:10 to obtain high-quality paired-end reads (clean reads). 

The reference genome index of *Carya illinoensis* was constructed using HISAT2 [54] version 2.0.5. The clean reads were mapped against the reference genome, and tables of mapped counts were obtained using FeatureCounts [55] version 1.5.0-p3.

### 4.5. Differential Expression Analysis

The tables of mapped read counts for each RNA-seq library were imported into edgeR [56] version 3.36.0 in the R session. Only genes that had achieved at least four counts per million (CPM) in at least four libraries were retained, and low-expressing genes were filtered. Differential expression analysis was performed as described in the study by Lim and colleagues [57]. A list of statistically significant differentially expressed transcripts with a false discovery rate (FDR) < 0.05 was exported for downstream analysis. The differential expression analysis for male and female flower buds at the same developmental stages (MD1 vs. FD1, MD2 vs. FD2, and MD3 vs. FD3) and male flower buds’ (MD2 vs. MD1 and MD3 vs. MD1) and female flower buds’ (FD2 vs. FD1 and FD3 vs. FD1) developmental stages were performed. The UpSetR [35] version 1.4.0 R package was utilized to visualize the composition of the datasets.

### 4.6. Genes Functional Analysis

The differentially expressed genes were subjected to GO enrichment analysis to better understand how the genes may be involved in floral development. The GO enrichment analysis of male and female flower buds at the same developmental stage and male and female flower buds’ development stages was realized using the R package clusterProfiler [58] version 4.2.2. The clusterProfiler performed enrichment analysis in which the gene length deviation was corrected. The GO-enriched DEGs with corrected *p*-values less than 0.05 were significantly enriched. To shed light on changes in biological functions during flower bud development from the transcriptome point of view, enriched GO biological process hierarchical structure relationship acyclic graphs were constructed using GOATOOLS [40] version 1.2.3. After the GO enrichment analysis, the DEGs were subjected to the KEGG pathway enrichment analysis to understand the pathways involved during flower bud development using the R package clusterProfiler [58] version 4.2.2. 

GSA was performed using the R package PIANO [39] version 2.10.1 to study global changes in early male and female flower buds. The logarithm of the fold change value, the *p*-values of the early stages of DGEs in male and female flower buds, and the GO terms were used for the GSA analysis with default parameters in the PIANO R session.

### 4.7. Analysis of Gene Correlation and Expression Trend

From the transcriptome point of view, genes related to the reproductive process were obtained from the GSA and GO enrichment analysis results to shed light on changes related to female and male flower bud differentiation. GO biological functional hierarchical relational graphs were constructed using GOATOOLS [40] version 1.2.3. The transcripts per million (TPM) values of these genes were transformed by log (TPM + 1) and averaged, and the correlation between genes was calculated using the Pearson method of the *cor* function. The results were visualized using the R package Pheatmap version 1.0.12. To explore the expression trends of these genes in female and male flower buds, the TPM values of these genes at different stages were averaged, and the expression profiles were visualized using Excel.

### 4.8. Quantitative Real-Time PCR 

Quantitative real-time PCR (qPCR) was performed to learn the expression levels of the female and male flower bud samples from which the RNA-seq data were generated. Six genes of female and male flower buds were randomly selected for qPCR. First-strand cDNA synthesis was performed on a total of 1 µg of total RNA of each female and male flower bud sample using the PrimeScript^™^ RT Reagent Kit with gDNA Eraser (Takara, Shiga, Japan) according to the manufacturer’s protocol. Primers (Supplementary Table S3) were designed using Primer6 software. The PCR reaction mixture was prepared using SYBR Green^™^ Realtime PCR Master Mix (TOYOBO, Osaka, Japan) with 10× diluted cDNA as a template, according to the manufacturer’s protocol. The qPCR was performed in a CFX96 Touch real-time PCR detection system (Bio-Rad, Hercules, CA, USA) with a program of 95 °C for 30 s, 40 cycles of 95 °C for 5 s, 58 °C for 10 s, and 72 °C for 15 s. Relative expression levels were calculated using the 2^−ΔΔCT^ method [59]. Gene actin was used as the reference gene. All reactions were performed using four biological samples with two technical replicates.

## 5. Conclusions

In this study, we determined the developmental stages of the female and male flower buds at higher resolution utilizing paraffin sectioning. Our transcriptome results suggest that the flowering pathway integrators *FT* and *SOC1* may play a role in the differentiation of the female and male flower buds of pecan. At the early stage of female and male flower bud differentiation, *STM* promoted the process of transition from leaf bud to flower bud, and *NF-YA1* facilitated flower transition. It was properly controlled by genes such as *CRY2* and *CIB1* to promote flower bud development in response to photoperiods. When the female and male flower primordia were formed, the characteristics of the female and male flower buds were determined. Gene correlation and expression trend analysis indicated that *J3*, *CRY2*, *CIB1*, *MEE14*, *MEE9*, and *MAPK6* played roles in female flower development. Among them, *J3* was highly expressed in the early stage of female flower bud development and adequately involved in regulating flowering time. Our results also suggest that *NF-YA1*, *PIF3*, *DTX35*, and *GAUT14* have roles in male flower bud development; for example, members of the NF-Y transcription factor family initiate downstream events leading to floral transformation. In addition, some genes, such as *AP2*, may play a role in establishing floral meristem characteristics and determining floral organ characteristics. Our results lay a foundation for the subsequent regulation of male and female flower buds, controlling the differentiation of male and female flower buds and increasing yield.

## Data Availability

The data presented in this study are available upon request from NCBI (https://www.ncbi.nlm.nih.gov/, accessed on 21 May 2022), accession number SAMN28512135- SAMN28512140.

## Supplementary Materials

The following are available online at https://www.mdpi.com/article/10.3390/plants12061378/s1, Figure S1: The results of qPCR and the expression trend of RNA-seq; Figure S2: Statistics of differential expression analysis results; Figure S3: Hierarchical relationship graph of differentially expressed genes in the male flower bud (MD1) during the initial stage of flower bud differentiation (FD1 vs. MD1); Figure S4: Hierarchical relationship graph of differentially expressed genes in the female flower bud (FD1) during the initial stage of flower bud differentiation (FD1 vs. MD1); Figure S5: Hierarchical relationship graph of differentially expressed genes in the male flower bud (MD2) during the flower primordial formation stage (FD2 vs. MD2); Figure S6: Hierarchical relationship graph of differentially expressed genes in the female flower bud (FD2) during the flower primordial formation stage (FD2 vs. MD2); Figure S7: Hierarchical relationship graph of the differentially expressed genes of MD2 vs. MD1; Figure S8: Hierarchical relationship graph of the differentially expressed genes of MD3 vs. MD1; Figure S9: Hierarchical relationship graph of the differentially expressed genes of FD2 vs. FD1; Figure S10: Hierarchical relationship graph of the differentially expressed gene enrichment of FD3 vs. FD1; Figure S11: Workflow of paraffin section experiment; Table S1: Statistics of pecan female and male flower buds transcriptome data; Table S2: Mapping rate of pecan female and male flower buds transcriptome data; Table S3: The primers and qPCR results and the expression trend of RNA-seq; Table S4: Differential expression genes of flower buds at different stages; Table S5: Gene set analysis of the differentially expressed genes during the initial differentiation of flower buds (FD1 vs. MD1) and the formation of flower primordial (FD2 vs. MD2); Table S6: GO enrichment analysis and KEGG pathway analysis of differentially expressed genes during flower bud differentiation stage (FD1 vs. MD1). Male flower bud (MD1) enriched; Table S7: GO enrichment analysis and KEGG pathway analysis of differentially expressed genes during flower bud initial differentiation stage (FD1 vs. MD1). Female flower bud (FD1) enriched; Table S8: GO enrichment analysis and KEGG pathway analysis of the differentially expressed genes during the formation of flower primordial (FD2 vs. MD2). Male flower bud (MD2) enriched; Table S9: GO enrichment analysis and KEGG pathway analysis of the differentially expressed genes during the formation of flower primordial (FD2 vs. MD2). Female flower bud (FD2) enriched; Table S10: GO enrichment analysis and KEGG pathway analysis of the differentially expressed genes in the formation of male flower primordial compared with the initial differentiation of flower buds (MD2 vs. MD1; Table S11: GO enrichment analysis and KEGG pathway analysis of the differentially expressed genes in the stamen primordia compared with the initial differentiation of flower buds (MD3 vs. MD1); Table S12: GO enrichment analysis and KEGG pathway analysis of the differentially expressed genes during the formation of female flower primordial compared with the initial differentiation of flower buds (FD2 vs. FD1); Table S13: GO enrichment analysis and KEGG pathway analysis of the differentially expressed genes in the pistil primordium compared with the flower bud differentiation (FD3 vs. FD1); Table S14: TPM values of candidate genes related at different stages of flower bud development
