# Supplementary figures and images for: Transcriptional Regulation of Female and Male Flower Bud Initiation and Development in Pecan (Carya illinoensis)
Source: Plants (Basel). 2023 Mar 20;12(6):1378. doi: 10.3390/plants12061378 (PMC10051282; doi:10.3390/plants12061378)

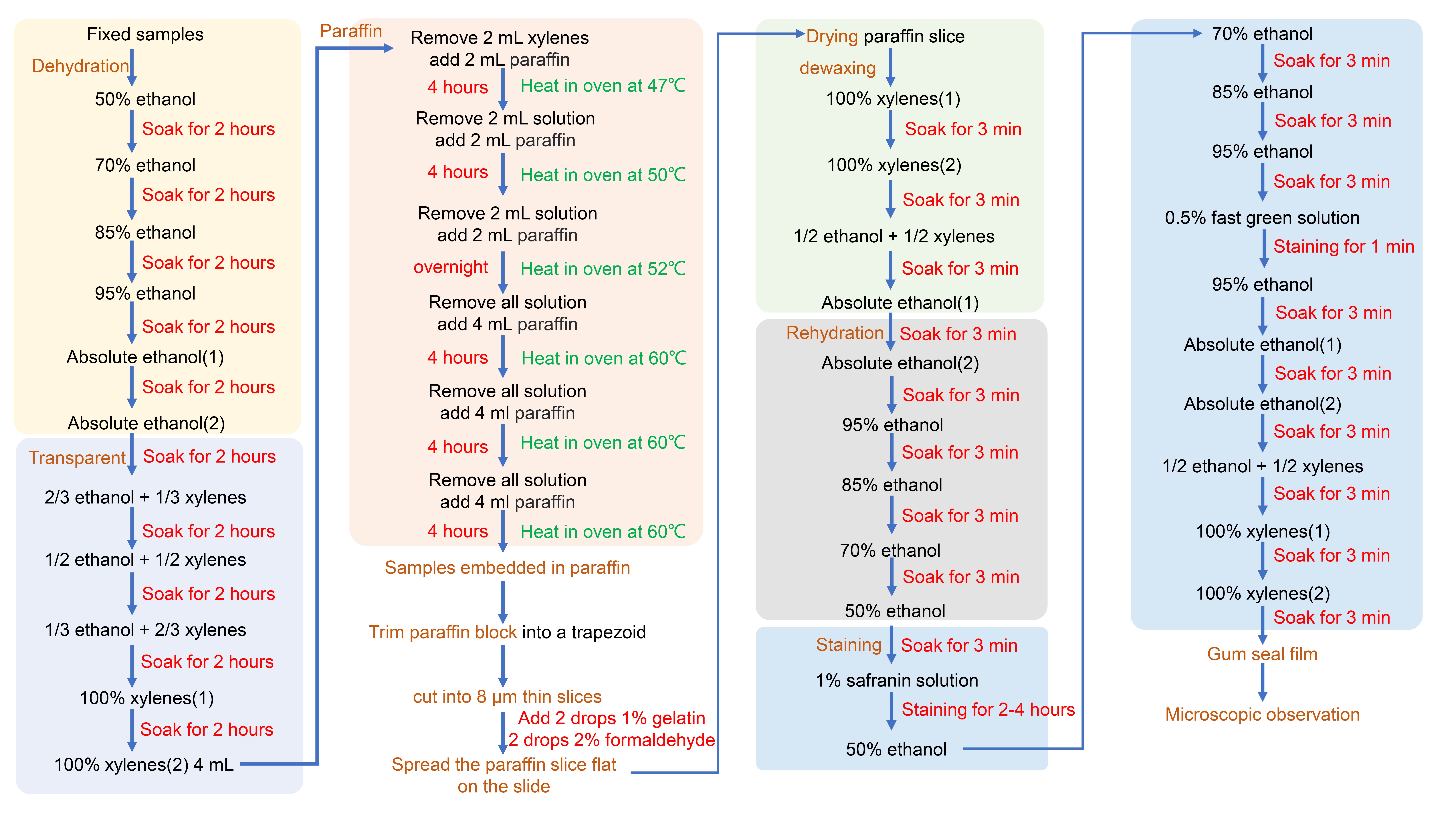

Supplement: Supplementary file 1 [file plants-12-01378-s001.zip › supplementaryMaterials/Supplementary_Figures/Supplementary Figure S11.jpg]

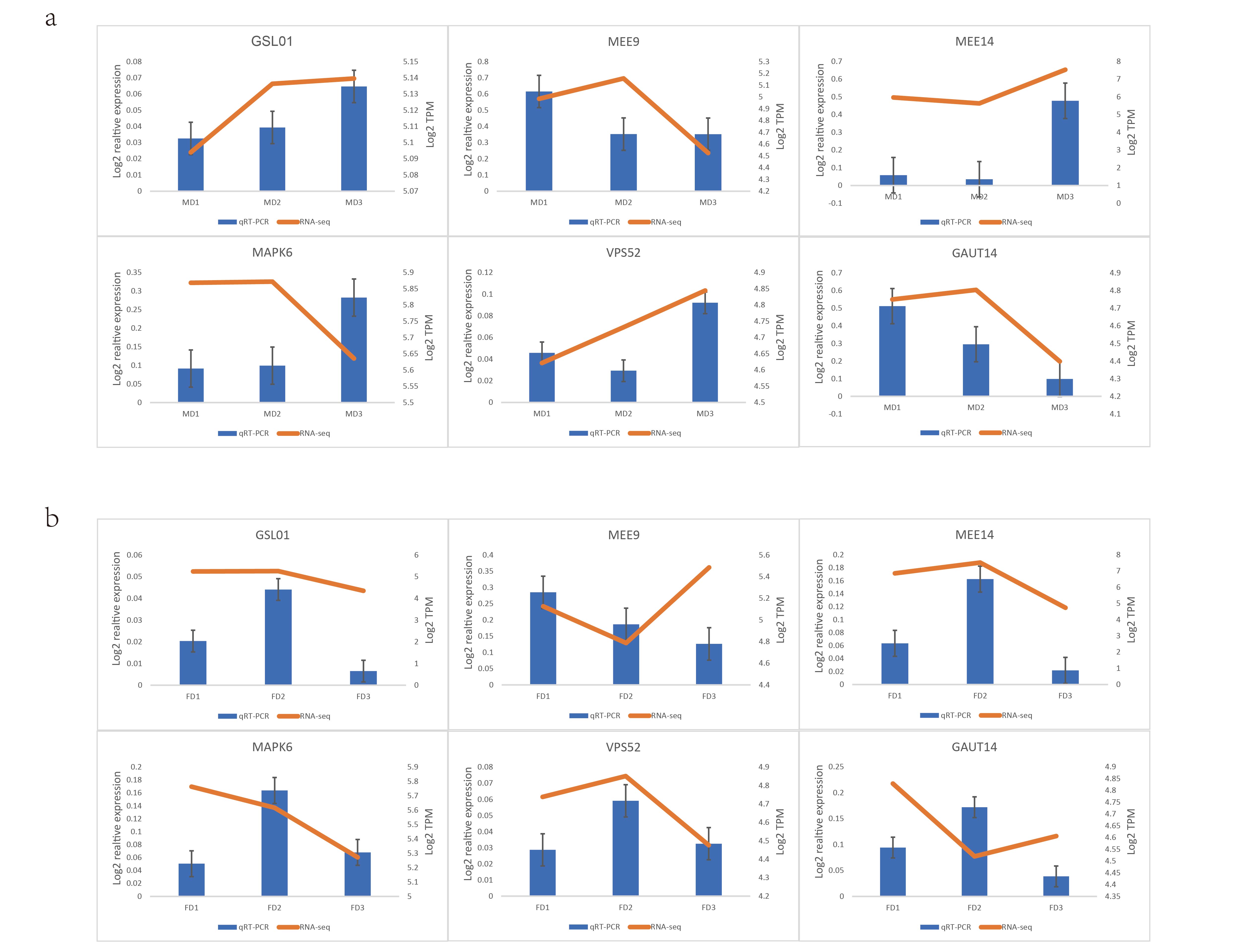

Supplement: Supplementary file 1 [file plants-12-01378-s001.zip › supplementaryMaterials/Supplementary_Figures/Supplementary Figure S1.jpg]

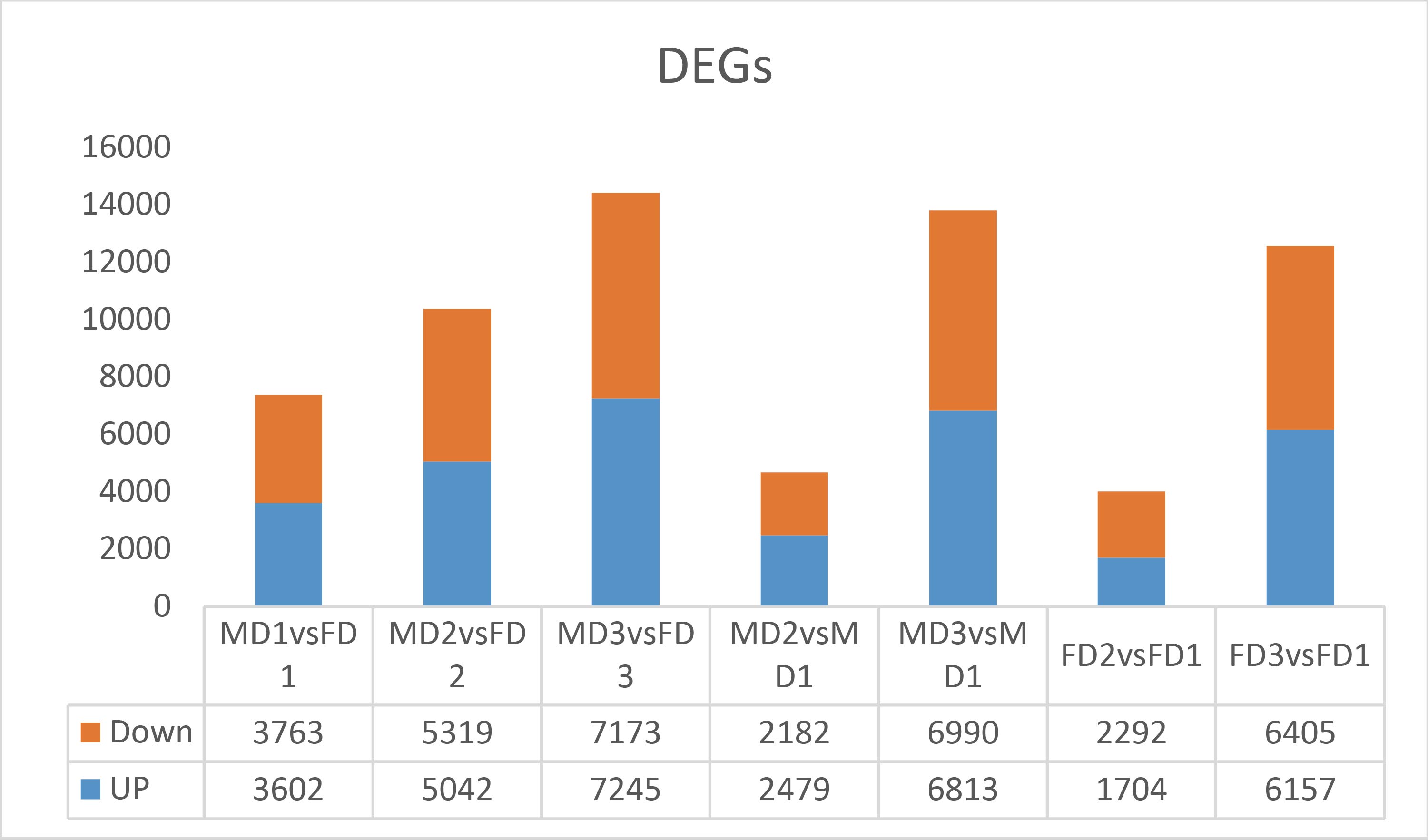

Supplement: Supplementary file 1 [file plants-12-01378-s001.zip › supplementaryMaterials/Supplementary_Figures/Supplementary Figure S2.jpg]
